# Precision-cut tumor tissue slices, a novel tool to study the tumor microenvironment interactions with chimeric antigen receptor (CAR) T cells

**DOI:** 10.1371/journal.pone.0327322

**Published:** 2025-08-08

**Authors:** Valeria Durante, Alina Wittwer, Benjamin Theek, Manuel Martinez-Osuna, Emmanuel Donnadieu, Olaf Hardt, Dominik Eckardt, Andreas Bosio, Sonja Schallenberg, Christoph Herbel

**Affiliations:** 1 Research and Development Department, Miltenyi Biotec B.V. & Co. KG, Bergisch Gladbach, Germany; 2 Université Paris Cité, CNRS, Inserm, Institut Cochin, Equipe Labellisée Ligue Contre le Cancer, Paris, France; Purdue University, UNITED STATES OF AMERICA

## Abstract

Up until present day, chimeric antigen receptor (CAR)-T cell therapy has only been approved for hematological malignancies, as CAR-T cells do not show comparable efficacy in solid tumors. Therefore, understanding the features of the tumor microenvironment (TME), is key to improve efficacy of adoptive cell therapies (ACTs) against solid tumors. In this context, robust workflows, which dissect the complex interactions between CAR-T cells and the TME are still lacking. To address this need, we have established an *ex vivo* workflow co-culturing tissue slices from patient tumor resections with CAR-T cells. The workflow is composed of assessing several complementary attributes, such as cytokine release via flow cytometry, quantification of cell infiltration into the tumor and assessment of the regions of the tissue slice the CAR-T cell infiltrate into by using the MACSima™ imaging cyclic staining technology. Using this workflow it is possible to observe the behavior of CAR-T cells within the tumor and its TME, their infiltration into distinct tumor compartments, as well as to dissect the underlying molecular mechanisms that drive T cell migration, thanks to MACSima™ multiplexing technology and its ability to image several markers at the same time. Assessment of ovarian carcinoma tissue slices revealed substantial release of specific cytokines and increased infiltration of T cells in the tumor areas when CAR-T cells were added to the tissue slices as compared to non-engineered T cells. The establishment of this novel approach will enable researchers to better characterize the interaction between CAR-T cells and the TME. Tissue slices present an intrinsic heterogeneity, which is indeed an advantage compared to other *in vitro* models but can turn itself into complex results interpretation. Therefore, we recommend that any conclusion derived from this assay should be verified with complementary models.

## Introduction

The emergence of immunotherapies during the past decades has improved targeted cancer treatment approaches. Specifically, CAR-T cell therapies show high efficacy in hematological cancers and several of them have been approved by FDA and EMA since 2017 [1–10].

Although CAR-T cell therapy shows a promising approach for solid tumor treatment as well [11–14], in these settings they did not show high efficacy [15,16]. One of the main factors for the reduced performance of CAR-T cells against solid tumors, is believed to be the tumor-microenvironment (TME), which includes the non-tumor cells, stroma and molecules surrounding the tumor cells.

So far, an effective approach to investigate the influence of the TME on individualized therapies has not yet been described. Here, we propose a workflow that enables researchers to directly explore the interaction of CAR-T cells with patient-derived tumor material in-depth.

For this reason, we focused on the tissue slice technique originally conceived in 1923 by Otto Warburg to study tumor metabolism [17,18]. Since its invention, the tissue slice technique has proven to be a versatile tool in many research fields, for metabolic and biochemical studies [19–21], neurophysiology [22,23], infectious diseases [24], and physiology [25–27].

Recently, tissue slices and similar models, like organoids and patient-derived tissue fragments (PDTFs), have been applied in screening assays for various diseases [24,28–30] with a particular focus on the oncology field in recent years [31–34]. Similarly to PDTFs and unlike organoids, tissue slices have the advantage of preserving all cellular and structural components of the TME as found in primary tumor. In addition, for the tissue slices no tumor-specific protocol is needed to setup the cultures. At the same time, using tissue slices enables to have a more comprehensive overview of the TME compartments and their respective organization in one specimen compared to PDTFs, as the size of the tissue slices is larger. Moreover, compared to the other abovementioned models, precision-cut tissue slices are prepared after embedding the sample in a solution of agarose dissolved in PBS, thus avoiding the criticality linked to the undefined composition and batch-to-batch variation of Matrigel® [35–37]. Therefore, we sought to employ tissue slices to study the interaction of CAR-T cells with the TME.

Several approaches have been described for preparing primary tumor material to establish *ex vivo* cultures and investigate features of the TME [28,30,38–40]. In these procedures, the main focus was to study basic disease development, the effects of previous treatments or the migratory behavior of CAR-T cells *ex vivo*. With the workflow described here, we can now further extend on previous application and explore the molecular underpinning driving CAR-T cells responsiveness towards primary patient material, as well as their distribution and interaction with different cellular subtypes in the TME.

In addition, a characterization of tumor specific markers expressed by individual tumors is also possible [41], opening up the potential for personalized screening of both plausible therapeutic targets and therapeutic options for the tumor material from a specific patient. Consequently, this assay might support decision-making for individualized therapies in the future.

Precision-cut tissue slices are a powerful tool to study the TME of solid tumors and its interaction with small molecules and cellular therapies, as cellular and architectural components that are present in the tumor can be preserved. However, great caution should be used while interpreting the results as, unlike cellular models and organoids, tumor tissue slices are not as easily standardizable.Their randomization when prepared from different specimen portions is a challenge, and they are more prone to experimental bias and selection of sample portions lacking completely either tumor or stroma compartments due to the intrinsic tissue heterogeneity. To mitigate these issues and have the highest possible homogeneity in the prepared tissue slices, a close collaboration with a pathologist to precisely identify the tumor and stroma portions in the specimen before tissue slices preparation is highly recommended, as well as including an adequate number of assay readout repetitions from tumor material derived from different patients. Moreover, it would be difficult to introduce genetic modifications to the TME of the tissue slices to verify experimental hypotheses, as done with cellular models and organoids. Finally, the introduction of artifacts created by means of the slicing procedure, such as creation of artificial distances due to the agarose solution penetration, must be taken into account when analyzing and interpreting the results, especially if performing distance analysis on the images. Therefore, to verify the findings observed with the tissue slices, we recommend to use complementary experimental models where parameters can be more easily controlled.

## Materials and methods

The study was conducted in accordance with the Declaration of Helsinki, and approved by the Ethics Committee of Ärztkammer Nordrhein (protocol code 2024026, approval date 29th of May 2024). Written informed consent was obtained from all subjects involved in the study.

After receipt of a tumor biopsy, a small part was dissociated to assess the sample composition, while another part was used to prepare precision-cut tumor tissue slices. This part of the tumor material was embedded in 5% (w/v) low gelling agarose (in PBS), and then cut to 400 µm slices using the Krumdieck Alabama R&D MD6000 Tissue Slicer (TSE Systems, Bad Homburg, Germany).

**Fig 1:**
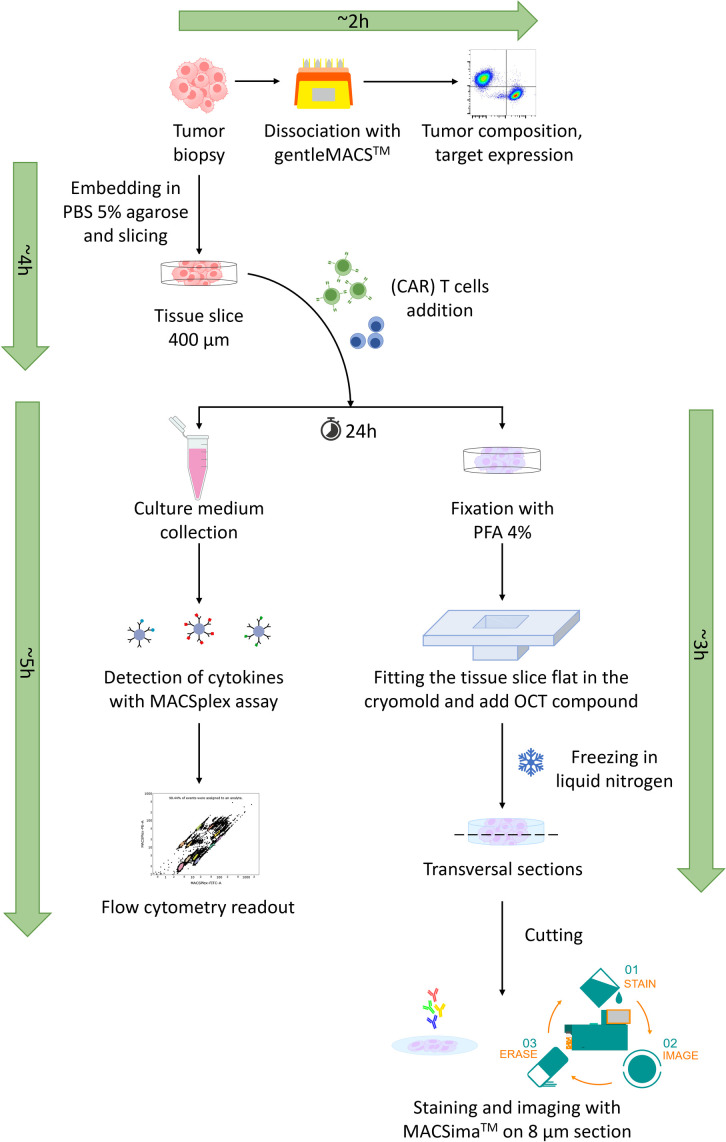
Overview of the workflow with the tissue slices. Tumor biopsies are split and either dissociated with the GentleMACS™ Octo Dissociator into a single cell suspension for quantitative analysis via flow cytometry, or embedded in agarose to prepare tissue slices of 400 μm thickness to set up the co-cultures. Every 24 hours during the co-culture, aliquots of the co-culture medium are withdrawn and stored at −20 °C for subsequent cytokine detection, while tissue slices are fixed in PFA 4% and kept at 4 °C. The tissue slices are then frozen to next prepare sections of 8 µm, which can be used with the MACSima™ System for the imaging of cyclic stainings on the tissue slices sections.

Subsequently, co-cultures of tissue slices with CAR-T cells were set up. As controls, co-cultures of tissue slices with untransduced (UTD) T cells from the same donor of the CAR-T cells and tissue slices without any cell addition were set up. To reduce experimental variability, the same batch of UTD and CAR-T cells was used for experiments with different patient material. The co-cultures were performed up to 96 hours (as also reported in [39]), while sampling every 24 hours the co-culture medium, to assess cytokine secretion, and tissue slices, to further investigate on cell composition and infiltration. For this scope, the tissue slices were fixed in 4% PFA, frozen in liquid nitrogen, and stored at −70 °C, for subsequent preparation of 8 µm sections for spatial multiplexed imaging with the MACSima™ System. An overview of this workflow is summarized in Fig 1.

Patient samples were collected from the 3^rd^ of June 2024 until the 18^th^ of December 2024. Written informed consent was obtained from all subjects involved in the study.

The protocol described in this peer-reviewed article is published on protocols.io (http://dx.doi.org/10.17504/protocols.io.rm7vz7qx8lx1/v1) and is included for printing as supporting information S1 File with this article.

## Expected results

**Fig 2:**
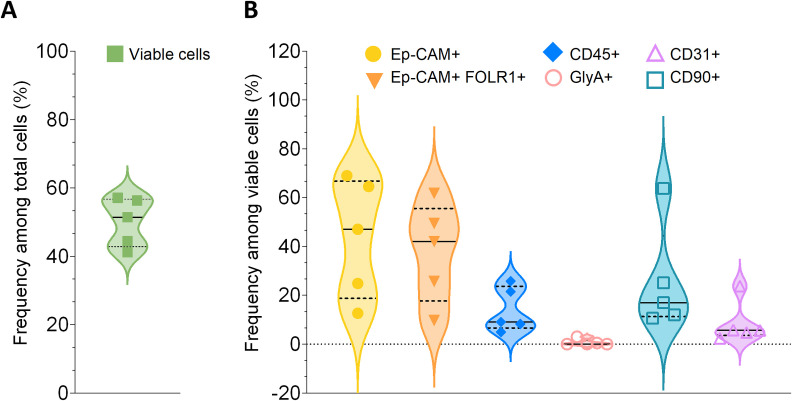
Composition of human ovarian cancer samples after dissociation. Five high grade serous ovarian carcinoma (HGSOC) samples (stage III) were freshly dissociated and stained via flow cytometry for dead cells, Ep-CAM cells (used as a surrogate tumor marker), folate receptor 1 (FOLR1, *CAR-target*), CD45 (leukocytes), Glycophorin-A (GlyA, erythrocytes), CD31 (endothelial cells), and CD90 (fibroblasts) before starting with tissue slices preparation. (A) The percentages of total viable cells (green) in the samples are represented in the graph. (B) The percentages of Ep-CAM^+^ (yellow), Ep-CAM^+^ FOLR1^+^ (orange), CD45^+^ (blue), GlyA^+^(pink), CD90^+^ (teal), and CD31^+^ (lilac) cells among viable cells in the samples are represented in the graph. (A,B) Each dot represents an individual donor. Median (black continuous line) and quartiles (black dotted line) are shown. An example of the gating strategy used can be seen in **Supporting Information** **S1 Fig****. The raw data of the results shown in this figure are collected in Supporting Information S2 File**.

Before preparing the tissue slices, to have an idea of the sample composition we first dissociated a small part of the received biopsy with the Tumor Dissociation Kit human and the GentleMACS™ Octo Dissociator with Heaters, according to manufacturer's instructions. The obtained single cell suspension was stained and analyzed via flow cytometry to assess the cellular composition. Viability of cells and cellular composition after dissociation of five human high grade serous ovarian carcinoma (HGSOC) stage III samples are shown in Fig 2. Our small sample cohort was showing on average 50.1% of cell viability among total cells (Fig 2A, Supporting Information S2 File), and heterogeneity in the single cell populations of interest, having for example the Ep-CAM^+^ population ranging from 12.7% to 68.9% among viable cells and CD45^+^ population ranging from 4.9% to 25.8% among viable cells (Fig 2B, Supporting Information S2 File).

**Fig 3:**
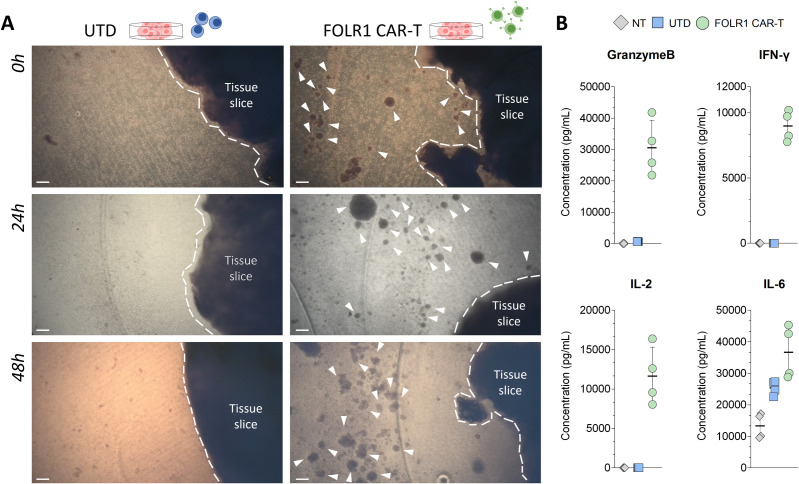
Activation of CAR-T cells on tissue slices. (A) Bright field images of ovarian carcinoma (HGSOC stage III) tissue slices co-cultured with 5 × 10^5^ total T cells, either untransduced (UTD) T cells or FOLR1 CAR-T cells from the same donor (left and right side, respectively), during a time course of 48 hours (top-down). Borders of the tumor tissue within the tissue slices are highlighted by the white dotted line, while clusters of activated T cells are marked by white arrowheads. Scale bar: 182 µm. (B) Granzyme B, IFN-γ, IL-2, and IL-6 secretion after 24h in the co-culture medium of tissue slices where no cells (NT, grey), UTD T cells (light blue) or FOLR1 CAR-T cells (light green) were added. Each point represents a technical replicate. Mean values (black line) with SD are shown. **The raw data of the results shown (B) are collected in Supporting Information** **S2 File**.

During the co-culture assay, activated T cells are expected to form clusters within the first 24h [42–44]. At the start of the co-cultures, CAR-T cell clusters are formed rapidly. During a co-culture of high grade HGSOC tissue slices with CAR-T cells directed against folate receptor 1 (FOLR1) [45], the anti-FOLR1-CAR-T (FOLR1 CAR-T) cells started to cluster after 24h and this cluster formation proceeded further after 48h (Fig 3A, white arrowheads). The levels of different cytokines and other soluble molecules can be analyzed in the medium of the co-cultures. After 24h of co-culture, Granzyme B, IFN-γ, and IL-2 were detected in the medium of tissue slices treated with FOLR1 CAR-T cells, while IL-6 was already detectable in untreated samples (NT), increased its levels with the addition of untransduced (UTD) T-cells, and its amount was further elevated in the presence of FOLR1 CAR-T cells (Fig 3B, Supporting Information S2 File).

**Fig 4:**
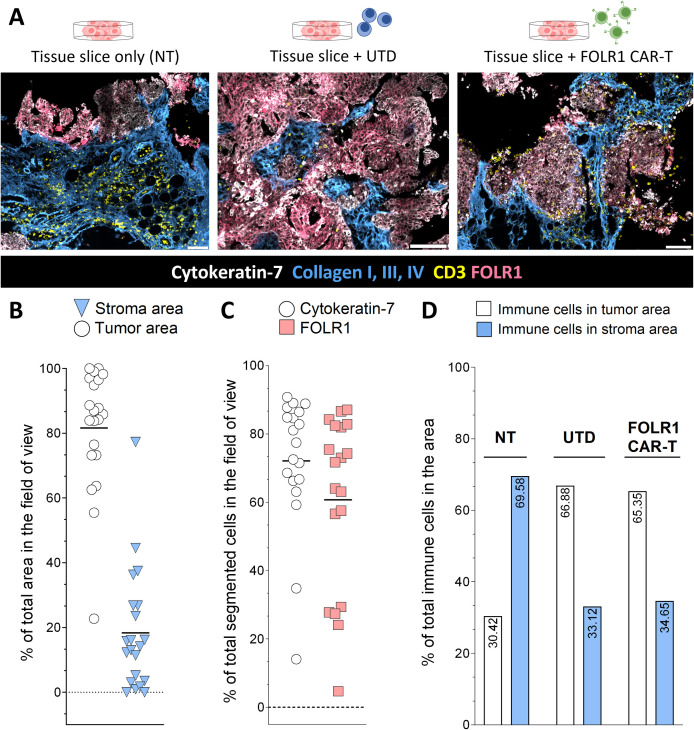
Exemplary compartments area and marker expression analysis from the co-culture assay of ovarian carcinoma tissue slices with 5 × 10^5^ total T cells, either untransduced (UTD) or FOLR1 CAR-T cells, after 24 hours of co-culture. (A) Representative images of four MACSima^TM^ experiments from one donor, showing cytokeratin-7 (white), FOLR1 (*CAR-target*, coral), collagen type I, III, IV (light blue), and CD3 (yellow) expression in tissue slices without any cell addition (NT), with UTD T cells or FOLR1 CAR-T cells addition. Scale bar: 100 µm. (B) Quantification of tumor (white) and stroma areas (light blue) in tissue slices without any cell addition (NT), with UTD T cells or FOLR1 CAR-T cells addition in several fields of view from four MACSima^TM^ experiments. (C) Quantification of cytokeratin-7 (white) and FOLR1 (coral) positive cells in several fields of view in tissue slices without any cell addition (NT), with UTD T cells or FOLR1 CAR-T cells addition from four MACSima^TM^ experiments. (D) Localization of immune cells in tumor (white) and in stroma (light blue) areas from four experiments in tissue slices without any cell addition (NT), with UTD T cells or FOLR1 CAR-T cells addition. Immune cells were identified via CD3, CD11c, and CD163 staining. The percentage of total immune cells in the different areas is written in black inside the bars. (B,C) Each dot represents the value in a field of view. Black line represents the mean. (C,D) Cells were identified after segmentation of the fields of view. (B,D) Tumor and stroma areas were identified respectively via positive cytokeratin-7 staining and via collagen type I, III, IV staining. **The raw data of the results shown in (B)-(D) are collected in Supporting Information** **S2 File.** **Corresponding H&E stainings of the samples shown in this figure are depicted in Supporting Information** **S2 Fig**.

**Fig 5:**
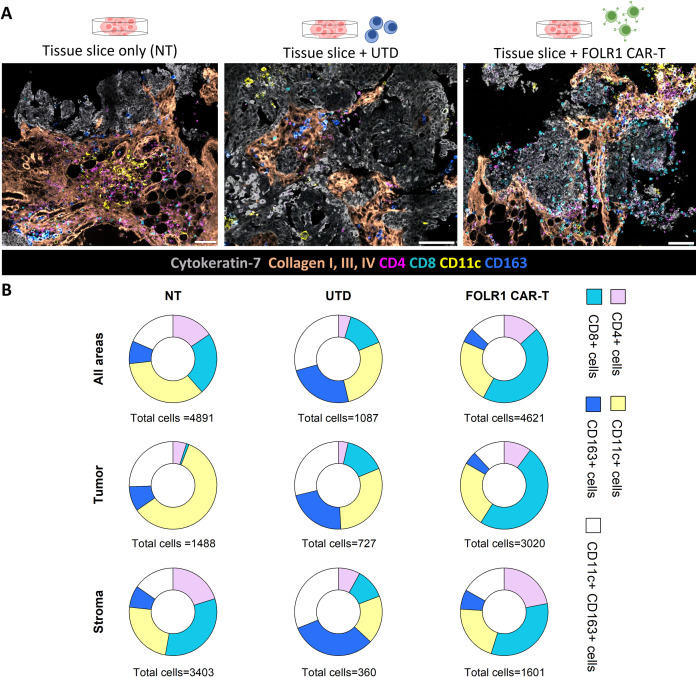
Exemplary immunophenotyping analysis from the co-culture assay of ovarian carcinoma tissue slices with 5 × 10^5^ total T cells, either untransduced (UTD) or FOLR1 CAR-T cells, after 24 hours of co-culture. (A) Representative images of four MACSima^TM^ experiments from one donor, showing cytokeratin-7 (grey), collagen I, III, IV (orange), CD4 (magenta), CD8 (cyan), CD11c (yellow), and CD163 (blue) expression in tissue slices without any T cell addition (NT), with UTD T cells, or FOLR1 CAR-T cells addition. Scale bar: 100 µm. (B) Quantification from four MACSima^TM^ experiments of the percentages of CD4^+^ (pink), CD8^+^ (cyan), CD11c^+^ (yellow), CD163^+^ (blue) and CD11c^+ ^CD163^+^ (white) cells in the whole sample (top row), in tumor (middle row) and in stroma areas (bottom row) in tissue slices without any cell addition (NT, left column), with UTD T cells (central column) or FOLR1 CAR-T cells (right column) addition. Cells were identified after segmentation of the fields of view. For UTD T and FOLR1 CAR-T treated tissue slices, percentages are related to the normalized tumor and stroma area. Tumor and stroma areas in UTD T and FOLR1 CAR-T treated tissue slices are normalized respectively to tumor and stroma areas of the NT tissue slices. Tumor and stroma areas were identified respectively via positive cytokeratin-7 and via collagen type I, III, IV staining. **The raw data of the results shown in this figure are collected in Supporting Information** **S2 File.** **Corresponding H&E stainings of the samples shown in this figure are depicted in Supporting Information** **S2 Fig.**

Additionally, spatial multiplexed analysis using the MACSima^TM^ imaging platform and the MACS® iQ View–Spatial Biology software enabled the determination of the area covered by stroma, tumor, and CAR-target-expressing cells, which was identified by the expression of collagen (type I, III or IV), cytokeratin-7 and FOLR1, respectively (Fig 4A, B and C; Supporting Information S2 File). Moreover, the localization of cells within stromal and tumor associated areas can be analyzed (Figs 4D, 5; Supporting Information File S2). In particular, cellular subtype distribution in different areas of the tissue slice can be determined and quantified (Fig 5B), as well as the proximity of different cell subsets.

The infiltrate of immune cells varied between the different co-culture conditions by cell composition and frequency. For instance, varying frequencies of CD8-expressing cells were observed in different conditions. The highest frequency of CD8^+^ cells was detected in the FOLR1 CAR-T treated samples, especially in the tumor area. On the other hand, in the NT sample the main population detected in the whole field of view and in the tumor area were CD11c^+^ cells, while in the stroma area CD8^+ ^cells were enriched. In contrast, in UTD-treated samples myeloid cells were the main population of infiltrating cells, with an increased predominance of CD163^+^ cells and CD11c^+^ CD163^+ ^cells. Overall, the addition of FOLR1 CAR-T cells had a major impact on the composition of the infiltrating immune cells in tumor areas rather than in stroma areas, while the addition of UTD T cells increased the myeloid cell infiltration in the whole tissue slice. We also observed a higher number of CD8^+^ and CD4^+^ cells in tissue slices treated with FOLR1 CAR-T cells despite starting with the same number of total cells, indicating either a reduced survival of UTD T cells in the tumor tissue slices compared to FOLR1 CAR-T cells or an increased persistence of FOLR1 CAR-T cells compared to UTD T cells.

Currently, there are promising results showing the correlation between the readouts from the tissue slice assays and *in vivo* studies [46], and specifically for our target we have previously shown our target efficacy *in vivo* [45]. In this regard, the IFN-γ increase shown in Fig 3B when FOLR1 CAR-T are added to precision-cut tissue slices was already observed *in vivo* [45], pointing out a possible translation that needs to be further investigated in the future.

With this innovative and physiologically relevant approach, it is possible to gain detailed knowledge about the interaction of CAR-T cells with the tumor, as well as with healthy tissues. Screenings of optimal CAR candidates in an *ex vivo* model reflecting the complex and heterogenous TME are enabled with this approach. In addition, the effects of small molecules and biologics such as checkpoint inhibitors or bispecific antibodies [33,47,48], as well as universal CAR-T cells and bispecific adapters [49–54], can be investigated with these co-culture assays and the subsequent spatial multiplexed analysis.

In this way, drug candidates can be selected based on assays performed in a model that has similar characteristics to the tumor in a patient, including an immune suppressive microenvironment. This approach may therefore increase the success rate of new drug candidates when transitioning from pre-clinical to clinical development.

Moreover, this approach employing a patient-derived model system and in-depth characterization provides not only an effective tool to improve pre-clinical drug development, but it may also foster personalized medicine by predicting the outcome of treatment options *ex vivo*. Indeed, a correlation between clinical response and assay readouts has been investigated with regards to organoids and PDTFs, unveiling a positive correspondence between clinical response and assay results [33,34,47]*.*This personalized screening approach may ultimately lead to a tailored, and consequently more effective, treatment for patients.

Nevertheless, the limitations of the assay must be taken into account when interpreting the results. As we did not intend to address long-term interactions of CAR-T cells with TME, which are in our case better addressed by our previous *in vivo* study [45], we have only tested co-cultures for up to 96h. However, there is already literature showing up to 3 weeks culture for brain tissue slices [55], and a perfusion system like the one that was already implemented for tissue slices [56] or the ones applied in organ-on-a-chips [57–60], could support a longer term co-culture of the precision-cut tissue slices with CAR-T cells, allowing the assay to address critical challenges in ACT such as exhaustion and persistence.

## Supporting information

S1 File Step-by-step protocol, also available on protocols.io http://dx.doi.org/10.17504/protocols.io.rm7vz7qx8lx1/v1.
(PDF)

S2 File Data from Figs 2, 3, 4, and 5. (XLSX)

S1 Fig Gating strategy for dissociated ovarian cancer tumor cells. (A) Pre-gating for ovarian cancer tumor samples, first identifying singlets, then tumor cells and finally viable and dead cells (left to right). A scheme of the gating is shown on the top part of the figure, while the corresponding density plot from an exemplary sample is shown at the bottom of the figure. Total cell percentages of the drawn gates are shown in black. (B) Gating of the desired cell populations (Ep-CAM^+^ , Ep-CAM^+^ FOLR1^+^ , CD45^+^ , Glycophorin-A (GlyA)^+^, CD90^+^ , and CD31^+^) from the viable cells. A scheme of the gating is shown on the top part of the figure, while the corresponding density plot from an exemplary sample is shown at the bottom of the figure. Total cell percentages of the drawn gates are shown in black.
(TIF)

S2 Fig H&E stainings of the sections shown in Figs 4 and 5. Hematoxylin and eosin stainings of the sections shown in Figs 4 and 5 from tissue slices without any cell addition (NT), with UTD T cells or FOLR1 CAR-T cells addition. The areas shown in Figs 4 and 5 are highlighted by the black line. Scale bar: 1 mm.
(TIF)
