# Supplementary material for: Precision-cut tumor tissue slices, a novel tool to study the tumor microenvironment interactions with chimeric antigen receptor (CAR) T cells
Source: PLoS One. 2025 Aug 8;20(8):e0327322. doi: 10.1371/journal.pone.0327322 (PMC12334022; doi:10.1371/journal.pone.0327322)
Supplement: S1 File — Step-by-step protocol, also available on protocols.io http://dx.doi.org/10.17504/protocols.io.rm7vz7qx8lx1/v1. (PDF) [file pone.0327322.s001.pdf]

Precision-Cut Tumor Tissue Slices as a Tool to Study the Tumor Microenvironment Interactions with Chimeric Antigen Receptor (CAR) T Cells

RESERVED DOI:
10.17504/protocols.io.rm7vz7qx8lx1/v1

Valeria Durante[1], Alina Wittwer[1], Benjamin Theek[1], Manuel Martinez-Osuna[1], Emmanuel Donnadieu[2], Olaf Hardt[1], Dominik Eckardt[1], Andreas Bosio[1], Sonja Schallenberg[1], Christoph Herbel[1]

[1]Research and Development Department, Miltenyi Biotec B.V. & Co. KG, Bergisch Gladbach, Germany;
[2]Université Paris Cité, CNRS, Inserm, Institut Cochin, Equipe Labellisée Ligue Contre le Cancer, Paris, France

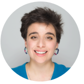

Valeria Durante
Miltenyi Biotec B.V. & Co. KG

Protocol Info: Valeria Durante, Alina Wittwer, Benjamin Theek, Manuel Martinez-Osuna, Emmanuel Donnadieu, Olaf Hardt, Dominik Eckardt, Andreas Bosio, Sonja Schallenberg, Christoph Herbel . Precision-Cut Tumor Tissue Slices as a Tool to Study the Tumor Microenvironment Interactions with Chimeric Antigen Receptor (CAR) T Cells. **protocols.io**
https://protocols.io/view/precision-cut-tumor-tissue-slices-as-a-tool-to-stu-e9bsbh2nf

Created: April 12, 2025

Last Modified: April 27, 2025

Protocol Integer ID: 160850

Keywords: CAR-T cells, TME, 3D models, precision-cut slices, PCS, MACSima™, ovarian carcinoma, HGSOC, ACT

Funders Acknowledgements:
Marie Skłodowska-Curie grant "T-OP" , European Union's Horizon 2020 research and innovation program
Grant ID: 955575

Disclaimer

Valeria Durante, Alina Wittwer, Benjamin Theek, Manuel Martinez-Osuna, Olaf Hardt, Dominik Eckardt, Andreas Bosio, Sonja Schallenberg and Christoph Herbel are or were employees of Miltenyi Biotec B.V. & Co. KG at the time of data collection.

Abstract

Up to date chimeric antigen receptor (CAR)-T cell therapy has been approved only for hematological malignancies, as CAR-T cells do not show comparable efficacy in solid tumors. Therefore, understanding the features of the tumor microenvironment (TME) is key to achieve improved efficacy of adoptive cell therapies (ACTs) against solid tumors. In this context, robust workflows dissecting the complex interactions between CAR-T cells and the TME are still lacking.

To address this need, we have established an ex vivo workflow co-culturing tissue slices from patient tumor resections with CAR-T cells. We analyzed cytokine release via flow cytometry and cell infiltration into the tumor and stroma regions of the tissue slices using the MACSima™ imaging cyclic staining technology. Using this workflow, it is possible to observe the behavior of CAR-T cells within the tumor and its TME, as well as their infiltration in distinct tumor compartments.

Assessment of ovarian carcinoma tissue slices revealed substantial release of specific cytokines and increased infiltration of T cells in the tumor areas when CAR-T cells were added to the tissue slices, compared to non-engineered T cells. The establishment of this novel approach will enable us to characterize the interaction between CAR-T cells and the TME. Nevertheless, as tissue slices present an intrinsic heterogeneity, we recommend the use of complementary models to verify the findings of this assay.

Attachments

PDF

PROTOCOL_Durante_Pre ...

499KB

Guidelines

A scheme of the whole workflow can be seen in **Fig 1.**

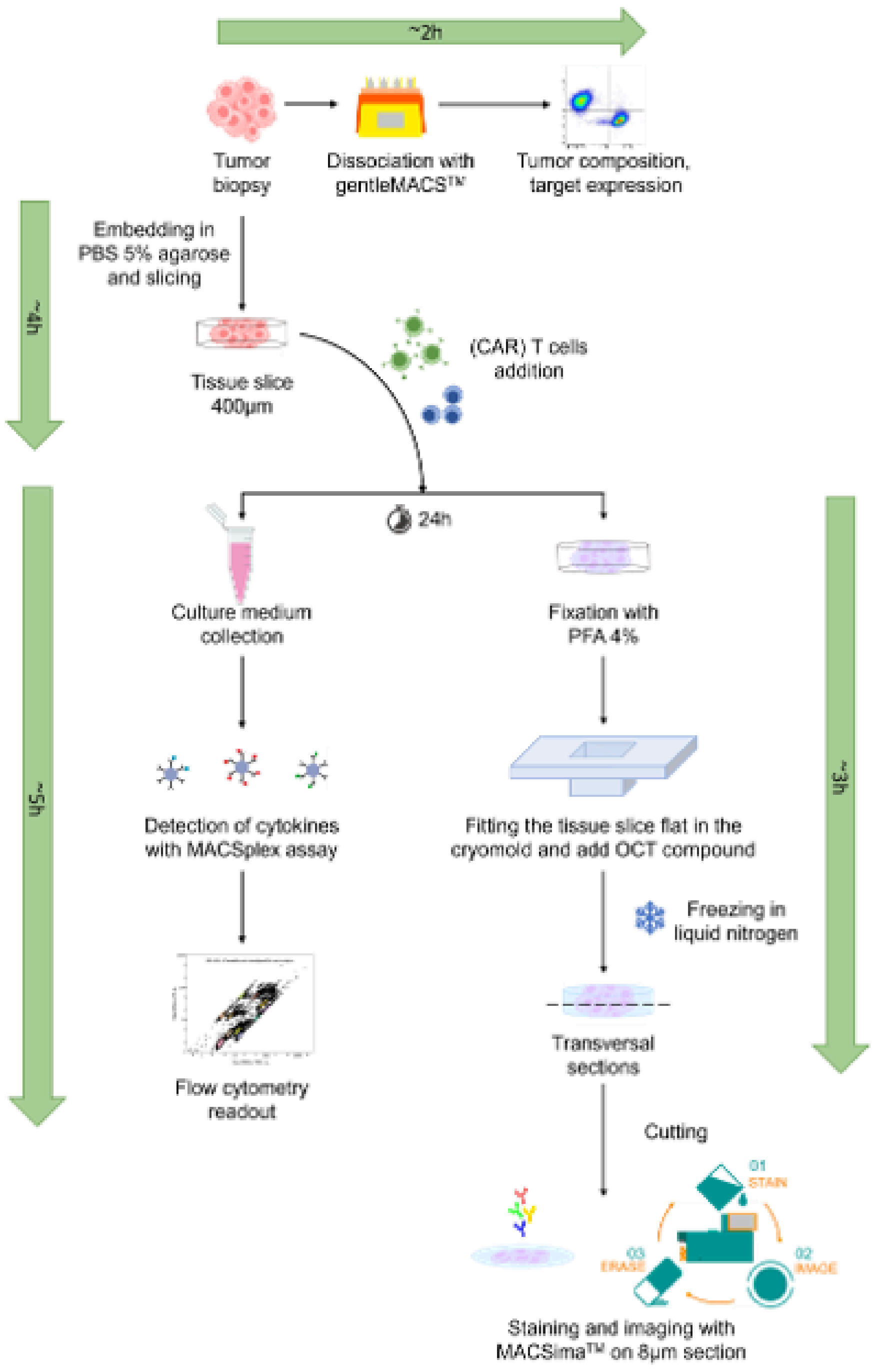

Fig 1. **Overview of the workflow with the tissue slices.** Tumor biopsies are split and either dissociated with the GentleMACS™ Octo Dissociator into a single cell suspension for quantitative analysis via flow cytometry, or embedded in agarose to prepare tissue slices of 400 µm thickness to set up the co-cultures. Every 24 hours during the co-culture, aliquots of the co-culture medium are withdrawn and stored at -20°C for subsequent cytokine detection, while tissue slices are fixed in PFA 4% and kept at 4°C. The tissue slices are then frozen to next prepare sections of 8 µm, which can be used with the MACSima™ System for the imaging of cyclic stainings on the tissue slices sections.

Materials

Materials

1. CAR-T cells and corresponding untransduced cells from the same donor to be tested. To reduce the experimental setup variability, we decided to use frozen cells of the same batch for tumor material coming from different patients.
2. Tumor material to be tested: this protocol can be applied to either mouse xenografts or human biopsies from different tumor types.
3. Single-use sterile scalpels for sample preparation. We used the disposable scalpels CUTFIX®, size 23 (B. Braun Deutschland; Cat. no.: 9409813).
4. MACS® Tissue Storage Solution (Miltenyi Biotec; Cat. no.: #130-100-008) or medium of choice for the storage of the tumor sample before tissue slices preparation.
5. Low gelling temperature agarose, type VII-A (Sigma-Aldrich; Cat. no.: A0701).
6. Phosphate Buffered Saline without calcium and magnesium (PBS) (VWR International bv, Leuven, Belgium) or any PBS solution without calcium and magnesium of choice.
7. 6 well cell culture plates of choice. We used the Corning® Costar® Ultra-Low Attachment Multiple Well Plate (Corning; Cat. no.: CLS3471). This multiwell format allowed us to place 3 slices in each well and to easily handle the tissue slices without breaking them.
8. Millicell® Standing Cell Culture Inserts, hydrophilic PTFE, pore size 0.4 µm, diameter 30mm (Millipore; Cat. no.: PICM03050), suited for 6 well plate. They protect the tissue slices from shear stress and allowed us to easily exchange the medium out of the insert without perturbing the co-cultures.
9. Culture media of choice for the T cells to allow their recovery after thawing. We used TexMACS™ Medium (Miltenyi Biotec; Cat. no.: #130-097-196) supplemented with 12.5 ng/mL IL-7 (Miltenyi Biotec; Cat. no.: #130-095-367), 12.5 ng/mL IL-15 (Miltenyi Biotec; Cat. no.: #130-095-760) and 3% (v/v) human AB serum (Capricorn Scientific, Ebsdorfergrund, Germany).
10. Culture media of choice for the co-cultures: this should be a media suitable for tumor cell growth. For better assay reproducibility, we used the serum-free medium Ovarian TumorMACS™ Medium (Miltenyi Biotec, Bergisch Gladbach, Germany; Cat. no.: 130-119-483). 100 U/mL of penicillin and 100 µg/mL of streptomycin were supplemented to the medium. CRITICAL STEP culture media can have a high impact on the experiment, as well as lot-to-lot differences for the human AB serum, if used. We saw also an impact of the type of antibiotic used. A broad spectrum antibiotic can have side effects on the cells.
11. (Optional) TransAct™ (Miltenyi Biotec; Cat. no.: #130-128-758) or any other reagent of choice to activate T cells. We used it to compare the activation of the (CAR) T cells when using an activation reagent with the activation by co-culture with tumor tissue slices.
12. Stainless steel washers, 5.3 mm inner diameter, 10 mm outer diameter (Bauhaus, Mannheim, Germany; Cat. no.:10826459). We used them to concentrate the added cells on the tumor tissue.
13. Cryomolds of choice for freezing of tissue slices. We used either TT Cryomold® Biopsy, square (10×10×5 mm) (Science Services, Munich, Germany; Cat. no.: 4565) or TT Cryomold® Intermediate, square (15×15×5 mm) (Science Services, Munich, Germany; Cat. no.: 4566) depending on the size and shape of the tissue slices.
14. Tissue freezing medium (Leica Biosystems, Deer Park, IL, USA; Cat. no.: 14020108926) as optimal cutting temperature (OCT) compound.
15. Formaldehyde solution 37 % (v/v) in water (Sigma-Aldrich; Cat. no.: 252549), to be diluted to get a 4% (v/v) in PBS solution.
16. Sucrose (Sigma-Aldrich; Cat. no.: S0389), for the preparation of a cryoprotectant solution of sucrose 30% (w/v) in PBS to be used before freezing of tissue slices.

17. Isopentane (Sigma-Aldrich; Cat. no.: M32631), for sample freezing in liquid nitrogen.
18. Microscopy slides of choice. We used the Epredia™ SuperFrost Plus™ Adhesion slides (Fisher Scientific, Schwerte, Germany; Cat. no.: 11950657).
19. Microtome blades of choice for the preparation of sections at the cryostat. We used the Epredia™ Ultra Disposable Microtome Blades (Fisher Scientific, Schwerte, Germany; Cat. no.: 12191830).
20. Cytokine detection reagents of choice. We used the MACSPlex Cytotoxic T/NK Cell Kit, human (Miltenyi Biotec, Bergisch Gladbach, Germany; Cat. no.: 130-125-800) and the MACSPlex Cytokine 12 Kit, human (Miltenyi Biotec, Bergisch Gladbach, Germany; Cat. no.: 130-099-169).
21. (Optional) Tumor Dissociation Kit, human (Miltenyi Biotec, Bergisch Gladbach, Germany; Cat. no.: 130-095-929) for tumor tissue dissociation.
22. (Optional) RPMI 1640 (Biowest, Riverside, MO, USA) for tumor tissue dissociation.
23. (Optional) GentleMACS™ C Tubes (Miltenyi Biotec, Bergisch Gladbach, Germany; Cat. no.: 130-093-237) for tumor tissue dissociation.
24. (Optional) MACS® SmartStrainers (70 µm) (Miltenyi Biotec, Bergisch Gladbach, Germany; Cat. no.:130-098-462) for tumor tissue dissociation.

Equipment

- Tweezers to manipulate the tissue slices. We found that curved tweezers worked best for us.
- Long tweezers for sample freezing in liquid nitrogen.
- Microtome suitable for living tissue slicing. We used the Krumdieck Alabama R&D MD6000 Tissue Slicer (TSE Systems, Bad Homburg, Germany). The use of a vibratome like the VT1200S (Leica Biosystems, Deer Park, IL, USA) is also possible.
- Laminar flow cabinet.
- CO_2 Incubator of choice.
- CM1860 UV Cryostat (Leica Biosystems, Deer Park, IL, USA) for the preparation of sections from the frozen tissue slices.
- MACSima™ System (Miltenyi Biotec, Bergisch Gladbach, Germany; Cat. no.: 130-121-164) for the cyclic imaging staining on the tissue slices.
- Flow cytometer of choice.
- GentleMACS™ Octo Dissociator with Heaters (Miltenyi Biotec, Bergisch Gladbach, Germany; Cat. no.: 130-134-029) for tumor tissue dissociation.

B Braun Cutfix® Disposable Scalpel Type 23 **Reagecon Diagnostics Ltd Catalog #**9409813

MACS® Tissue Storage Solution **Miltenyi Biotec Catalog #**130-100-008

Agarose, low gelling temperature **Merck MilliporeSigma (Sigma-Aldrich) Catalog #**A0701

Corning® Costar® Ultra-Low Attachment Multiple Well Plate **Merck MilliporeSigma (Sigma-Aldrich) Catalog #**CLS3471

Millicell Cell Culture Insert, 30 mm, hydrophilic PTFE, 0.4 µm, Hydrophilic PTFE **Merck MilliporeSigma (Sigma-Aldrich) Catalog #**PICM03050

TexMACS™ Medium **Miltenyi Biotec Catalog #**130-097-196

Human IL-7 **Miltenyi Biotec Catalog #**130-095-367

Human IL-15 **Miltenyi Biotec Catalog #**130-095-760

Ovarian TumorMACS™ Medium, human **Miltenyi Biotec Catalog #**130-119-483

- T Cell TransAct™ human **Miltenyi Biotec Catalog #**130-111-160
- Tissue-Tek® Cryomold® Cryomolds **Scienceservices Catalog #**4565
- Tissue-Tek® Cryomold® Cryomolds **Science services Catalog #**4566
- Tissue Freezing Medium **Leica Biosystems Catalog #**14020108926
- Formaldehyde solution **Merck MilliporeSigma (Sigma-Aldrich) Catalog #**252549
- Sucrose **Merck MilliporeSigma (Sigma-Aldrich) Catalog #**S0389
- 2-Methylbutane **Merck MilliporeSigma (Sigma-Aldrich) Catalog #**M32631
- Epredia™ SuperFrost Plus™ Adhesion slides **Fisher Scientific Catalog #**11950657
- Epredia™ Ultra Disposable Microtome Blades **Fisher Scientific Catalog #**12191830
- MACSPlex Cytokine Kits **Miltenyi Biotec Catalog #**130-125-800
- MACSPlex Cytokine 12 Kit, human **Miltenyi Biotec Catalog #**130-099-169
- Tumor Dissociation Kit human **Miltenyi Biotec Catalog #**130-095-929
- GentleMACS C tube **Miltenyi Biotec Catalog #**130-093-237
- MACS SmartStrainers (70 µm) **Miltenyi Biotec Catalog #**130-098-462

Equipment

**MACSima™ System**	NAME
MACSima™ System	TYPE
Miltenyi Biotec	BRAND
130-121-164	SKU
https://www.miltenyibiotec.com/US-en/products/macsima-system.html	LINK

Equipment	
GentleMACS™ Octo Dissociator with Heaters	NAME
Dissociator with Heaters	TYPE
Miltenyi Biotec	BRAND
130-134-029	SKU
https://www.miltenyibiotec.com/US-en/products/gentlemacs-octo-dissociator-with-heaters.html	LINK

Safety warnings

Please beware of the individual reagents and chemicals safety warnings provided by the manufacturers. Formaldehyde is flammable, a suspected carcinogen, and can cause skin and respiratory system irritation.

Ethics statement

The study was conducted in accordance with the Declaration of Helsinki, and approved by the Ethics Committee of Ärztkammer Nordrhein (protocol code 2024026, approval date 29th of May 2024). Written informed consent was obtained from all subjects involved in the study.

Before start

Autoclave the stainless steel washers and the small tweezers that will be used in contact with the tumor tissue slices.

Tumor Tissue Dissociation (~2h)

1

Note

Before preparing the tissue slices, to have an idea of the sample composition we first dissociated a small part of the received biopsy with the Tumor Dissociation Kit human and the GentleMACS™ Octo Dissociator with Heaters, according to manufacturer instructions. The use of other equipment and materials of choice for tumor dissociation is also possible.

Obtain fresh tumor samples and store it in MACS® Tissue Storage Solution at 2-8 °C until processing.

1.1 PAUSE STEP (Optional) The sample can be stored at 2-8 °C in MACS® Tissue Storage Solution up to 48:00:00 if necessary.

2 Proceed with tumor dissociation by preparing the enzyme mix in a gentleMACS™ C tube from the Tumor Dissociation Kit in RPMI based on the weight of the sample, as of manufacturer instructions, and by resecting a small portion (between 0.2 g and 1.0 g) of the received sample. Prepare the sample for dissociation by removing adipose, fibrous, and necrotic tissue and cutting it in small pieces of 2-4 mm.

3 Transfer the sample in the gentleMACS™ C tube where the enzyme mix was prepared, minding to have the sample well immersed in the enzyme mix, and close the C tube tightly.

4 Load the C tube upside down onto the sleeve of the GentleMACS™ Octo Dissociator, load the heater on top and start the appropriate program.

Note

Depending on the nature of the tumor, i.e. a "hard" fibrous tumor or a "soft" adipose tissue rich tumor, there are different recommended dissociation programs. In our case with human high grade serous ovarian carcinoma (HGSOC) samples, we were mostly employing the recommended program for soft tumors (37C_h_TDK_1), unless the sample looked very fibrous and stiff. In those cases, we employed the recommended program for medium tumors (37C_h_TDK_2).

5

- At the end of the dissociation, detach the C tube and pour the cell suspension in a falcon tube with a pre-wet MACS® SmartStrainers (70 µm).
- Wash the strainer with RPMI and centrifuge the cell suspension at 300 x g, Room temperature for 7-10 minutes.

6 Discard the supernatant and resuspend the cell pellet in flow cytometry staining buffer. Proceed with flow cytometry staining.

Tissue Slices Preparation and Co-Culture Set Up: Tissue slices preparation (~2h including all preparation steps)

10m

7 Obtain fresh tumor samples and store it in MACS® Tissue Storage Solution at 2-8 °C until processing.

Note

For tissue slices preparation, there is no need for large samples. From less than 1 g , approximately 20-25 slices can be obtained, which was enough to perform the co-culture with 3 conditions in duplicates.

7.1 PAUSE STEP (Optional) The sample can be stored at 2 °C - 8 °C in MACS® Tissue Storage Solution up to 48h if necessary.

8 Supplement the medium of your choice for the co-cultures under a laminar flow cabinet.

8.1 We prepared the Ovarian TumorMACS™ Medium according to the manufacturer's instructions and supplemented it with 100 U/mL of penicillin and 100 µg/mL of streptomycin. Also, supplement the medium for T cells recovery after thawing, if you use frozen cells. We used TexMACS™ Medium supplemented with 12.5 ng/mL IL-7, 12.5 ng/mL IL-15 and 3% (v/v) human AB serum.

9 In case frozen cells are used, thaw CAR T cells and corresponding untransduced cells from the same donor and allow them to recover in complete TexMACS™ Medium.

Note

If the tissue slices are prepared the day following to the sample delivery day, it is recommended to thaw the cells on the day of sample delivery to allow overnight recovery after thawing.

10 Assemble the Krumdieck Tissue Slicer according to manufacturer instructions and fill the reservoir assembly with cold medium.

11 Prepare sample in cubes of ~ 5 × 5 mm in cold medium with 2 scalpels to avoid shear stress to the sample when cutting. Keep On ice until embedding of the samples takes place.

12 Dissolve 1 g of agarose in 20 mL of sterile of Phosphate-buffered saline (PBS) to prepare the 5% (w/v) agarose solution and place it in the incubator at 37 °C for 00:05:00 to cool it down. 5m

13
- Close the bottom of the embedding cylinders of the Krumdieck Tissue Slicer with para-film.
- Pour the agarose inside the embedding cylinders up to ¾.
- Remove excess medium from the sample cubes using a tissue wipe and place the cube in the center of the embedding cylinder with the help of the curved tweezers while the agarose is still liquid.
- Cover with an additional low amount of agarose.

Note

CRITICAL STEP It is important to dry the excess medium from the sample, to prevent formation of bubbles in the agarose during the embedding, and to clean the tweezers from excess agarose after each sample. Not performing the above-mentioned steps can result in more fragile agarose and lead to the disruption of the tissue slices.

CRITICAL STEP Try to place the sample in the center of the cylinder, to have it well surrounded by the agarose. An example is shown in **Fig 2A**.

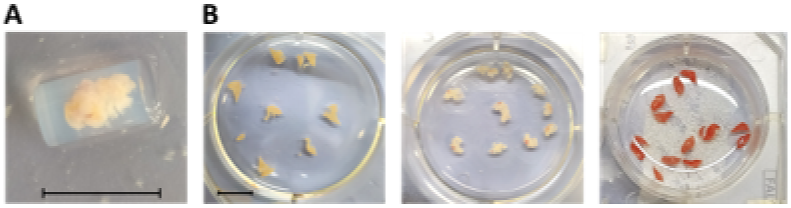

Fig 2. **Sample embedding in agarose and examples of tissue slices from different mouse organs.** (**A**) Sample embedded for tissue slicing, well surrounded by agarose. Scale bar: 10 mm. (**B**) Tissue slices prepared from different mouse organs (liver, ovarian cancer xenograft, lungs; left to right) with different stiffness characteristics. They were used to set up the optimal slicing conditions for the workflow with patient tumor biopsies. Scale bar: 8 mm.

14 Cool down the embedding cylinders for 00:05:00 On ice to allow agarose solidification. 5m

15
- Once the agarose is solidified, remove parafilm and pull out the agarose cylinder from the embedding cylinder using the back of the tweezers.
- Trim out excess agarose (leave ~1 mm at both ends).
- Insert the embedding cylinder in the Krumdieck Tissue Slicer, gently place the agarose cylinder with the tumor tissue inside it, and place the plunger with the weight on top of the agarose cylinder, to keep it at the bottom of the embedding cylinder while slicing.

16 Settle the adjustment knob to adjust the desired tissue thickness and the arm and blade speed to the desired settings. Start the slicing.

Note

We used 400 µm, arm speed to 2 out of 5 and blade speed to 3 out of 5.

17 Collect the tissue slices in a vessel by opening the pinch clamp of the Krumdieck Tissue Slicer, and transfer them in a 6-well plate with 1 mL cold PBS added to each well.
Keep the plate On ice until all slicing is performed.

Note

Ideally, the slices should look as shown in **Fig 2B**, with the tissue well placed inside the agarose.

CRITICAL STEP Use either the tweezers or a thin brush to move and collect the tissue slices, minding to be as gentle as possible. Consider training with test material to acquire the necessary manual dexterity before working with patient material.

Tissue slices preparation and co-culture set up: Co-Culture Set Up (~2h including all preparation steps)

1h

18 Prepare a 6-well plate with tissue culture inserts and add 1 mL of co-culture medium to the well and 500 µL to the insert.

19 Select the best tissue slices for the co-cultures by amount of tumor tissue embedded and integrity.

20 Transfer the tissue slices carefully into the cell culture inserts and wash them twice with medium for 00:30:00 inside the incubator.

30m

Note

In this step, we tried to distribute tissue slices obtained from different tumor biopsies portions by having one tissue slice prepared from the same biopsy portion represented in all experimental conditions.
CRITICAL STEP be very careful while handling the tissue slices, as they are fragile and can easily break.

21 Meanwhile prepare the cells needed for the experiment. Resuspend 1-5 × 10^5 UTD and/or CAR T cells per slice. Cell numbers are related to total T cell count. Adjust them based on the frequency of CAR+ cells) in 40 µL of the co-culture medium.

22 After washing of the tissue slices, take the plate out of the incubator and remove the medium from the insert. Place sterilized steel washers on top of each slice, minding to

have the tumor tissue inside the inner opening of the washers (**Fig 3**). This will be used to keep the added cells centered on the tumor tissue.

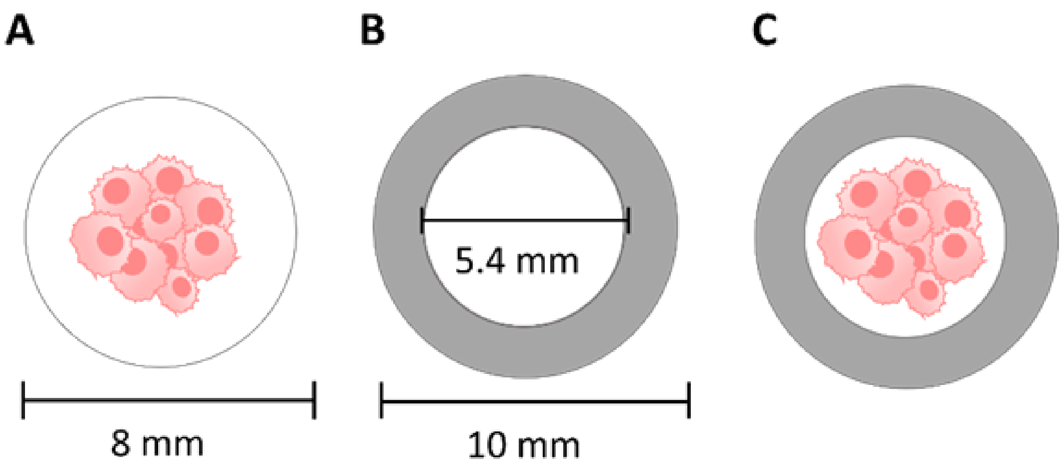

Fig 3. **Positioning scheme of the steel washer on top of the tissue slice within the culture plate.** (**A**) Diameter of a tissue slice is 8 mm. (**B**) The inner and outer diameter of a steel washer are 5.4 mm and 10 mm, respectively. (**C**) The steel washer should be positioned centered around the tissue slice.

23 30m

- Place 40 µL of cell suspension inside of the washer for each slice, and let them rest for 00:30:00 in the incubator.
- Afterwards, gently add 500 µL of medium dropwise on top of the tissue slices, and remove the steel washers.

Tissue slices preparation and co-culture set up: Co-Culture Maintenance and Sample Uptake (~1h) 35m

24 Remove 800 µL of medium from each well, and store it at -20 °C until detection of cytokines is performed.

25 OPTIONAL STEP In case you want to continue the co-culture for longer periods of time, add 800 µL of fresh pre-warmed medium into the well and gently add 500 µL dropwise on top of the tissue slices slice in the cell culture insert.

Note

In our hands, cultures of precision-cut slices were kept successfully up to 96:00:00, as also already reported in Misra *et al.*, 2019.

CITATION

Misra S, Moro CF, Del Chiaro M, Pouso S, Sebestyen A, Lohr M, et al (2019). Ex vivo organotypic culture system of precision-cut slices of human pancreatic ductal adenocarcinoma. Scientific Reports.
LINK
https://doi.org/10.1038/s41598-019-38603-w

26 Remove the tissue slices you want to investigate further and place them in a clean multi-well plate with PBS.

Note

CRITICAL STEP be very careful while handling the tissue slices, as they are fragile and can easily break.

27 Under a fume hood, add PFA 4% by gently pipetting on the side of the well, and incubate the tissue slices for 00:35:00 at 2-8 °C to fix them. 35m

28 Wash the tissue slices gently 3 times with PBS, remove all the PBS and add the 30% sucrose solution to the tissue slices. Store the tissue slices at 2-8 °C until freezing.

Preparation of Samples for Freezing and Sectioning for MACSima™ System Imaging 2d

29 Leave the 30% (w/v) sucrose solution until the tissue slices stop floating in the solution and sink to the bottom of the well, this can take up to 48:00:00. The aim is to avoid the formation of crystals when freezing the tissue slices. 2d

30 Label the cryomolds for the freezing of tissue slices and fill them with a layer of OCT medium at the bottom.

Note

CRITICAL STEP take care to avoid bubble formation with the OCT. Bubbles can burst during freezing and destroy the sample. Consider to remove bubbles with a pipette tip or similar.

31 Prepare a vessel with liquid nitrogen and a smaller vessel to be filled with the isopentane.

32 Dry the single tissue slices briefly using tissue paper, then place them flat in the cryomold on top of the OCT layer and add further OCT on top. Use a pipette tip to position the tissue slices in the OCT in a way that they have no creases and are placed at the bottom of the cryomold.

Note

CRITICAL STEP as aforementioned, mind to not create bubbles when adding the OCT. As in previous passages, be careful when handling the tissue slices.

33 Pre-cool the small vessel with isopentane in the bigger vessel with liquid nitrogen. Take the cryomolds with long tweezers and place them at the bottom of the small vessel with the isopentane. Keep the sample inside until the OCT changes its aggregation state, indicated by a change of color from transparent to white.

Note

CRITICAL STEP avoid to have liquid nitrogen getting in contact with the isopentane as their reaction leads to fixation of the cryomold to the bottom of the smaller vessel.

34 PAUSE STEP Store at -80 °C until further use.

35 Cut 8 µm sections at the cryostat for the subsequent MACSima™ System processing.

Note

Refer to the manufacturer's instructions for sample preparation before start of the run. The fixation step can be skipped, as our samples were already fixed in PFA 4%.

Protocol references

Misra, S., et al., Ex vivo organotypic culture system of precision-cut slices of human pancreatic ductal adenocarcinoma. Sci Rep, 2019. 9(1): p. 2133. https://doi.org/10.1038/s41598-019-38603-w

Citations

Step 25

Misra S, Moro CF, Del Chiaro M, Pouso S, Sebestyen A, Lohr M, et al. Ex vivo organotypic culture system of precision-cut slices of human pancreatic ductal adenocarcinoma
https://doi.org/10.1038/s41598-019-38603-w

Acknowledgements

Conceptualization: Valeria Durante, Emmanuel Donnadieu, Sonja Schallenberg, Dominik Eckardt, Andreas Bosio and Christoph Herbel
Formal analysis: Valeria Durante
Investigation: Valeria Durante
Methodology: Emmanuel Donnadieu, Valeria Durante, Alina Wittwer, Benjamin Theek, Manuel Martinez-Osuna
Supervision: Sonja Schallenberg, Dominik Eckardt, Andreas Bosio and Christoph Herbel
Visualization: Valeria Durante, Sonja Schallenberg and Benjamin Theek
Writing—original draft preparation: Valeria Durante and Sonja Schallenberg
Writing—review and editing: Alina Wittwer, Benjamin Theek, Emmanuel Donnadieu, Olaf Hardt, Andreas Bosio and Christoph Herbel

The authors would like to thank David Espie for technical support on tissue slices preparation, Fabio El Yassouri and Silvia Rüberg for the technical support on the MACSima™ System and the related sample preparation, Nicholas Tokarew for critically reading the manuscript and Prof. Dr. med. Christian Rudlowski and the patients from the Evangelisches Krankenhaus of Bergisch Gladbach.
