# Supplementary figures and images for: Precision-cut tumor tissue slices, a novel tool to study the tumor microenvironment interactions with chimeric antigen receptor (CAR) T cells
Source: PLoS One. 2025 Aug 8;20(8):e0327322. doi: 10.1371/journal.pone.0327322 (PMC12334022; doi:10.1371/journal.pone.0327322)

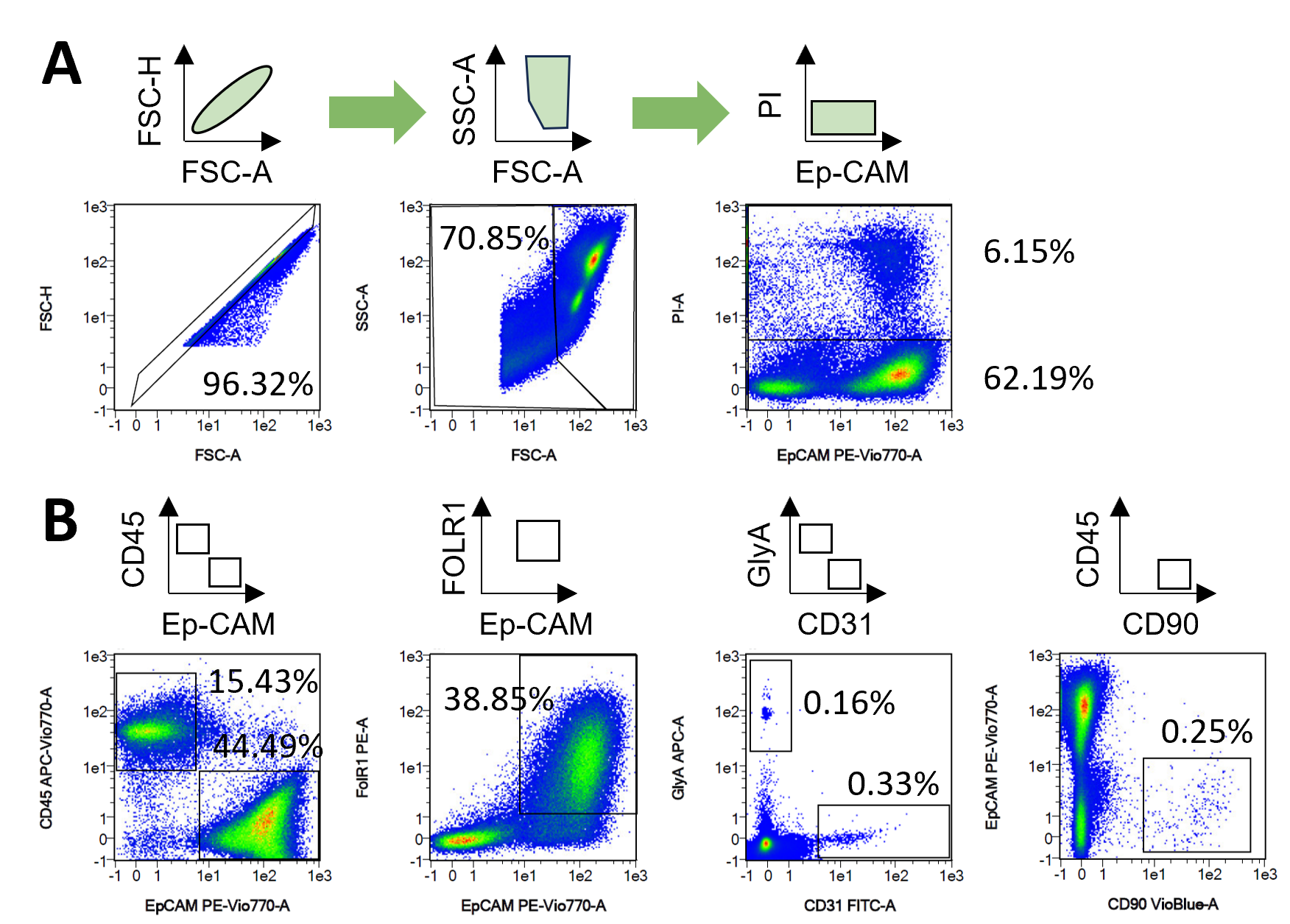

Supplement: S1 Fig — (A) Pre-gating for ovarian cancer tumor samples, first identifying singlets, then tumor cells and finally viable and dead cells (left to right). A scheme of the gating is shown on the top part of the figure, while the corresponding density plot from an exemplary sample is shown at the bottom of the figure. Total cell percentages of the drawn gates are shown in black. (B) Gating of the desired cell populations (Ep-CAM+ , Ep-CAM+ FOLR1+ , CD45+ , Glycophorin-A (GlyA)+, CD90+ , and CD31+) from the viable cells. A scheme of the gating is shown on the top part of the figure, while the corresponding density plot from an exemplary sample is shown at the bottom of the figure. Total cell percentages of the drawn gates are shown in black. (TIF) [file pone.0327322.s003.tif]

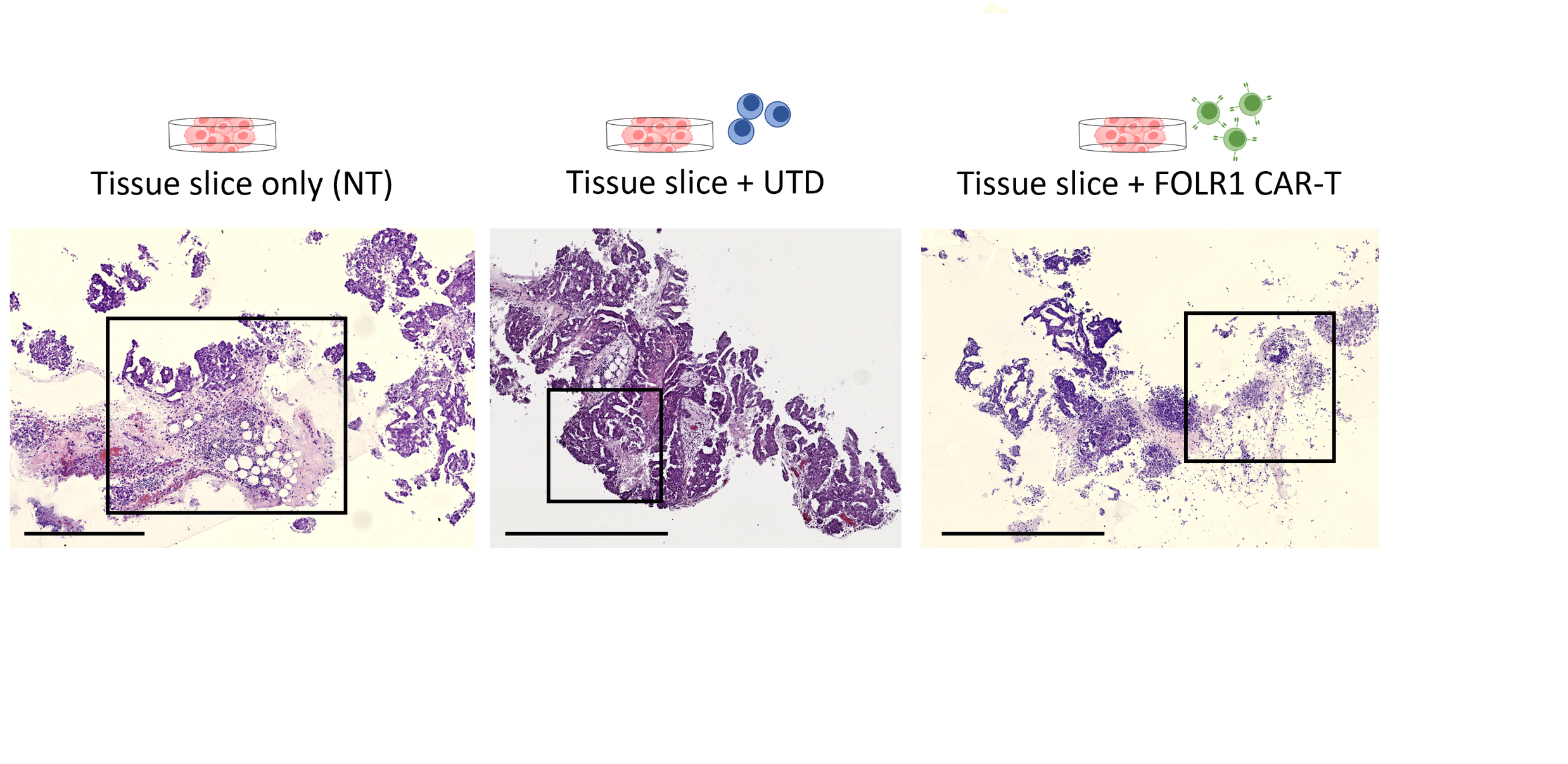

Supplement: S2 Fig — Hematoxylin and eosin stainings of the sections shown in Figs 4 and 5 from tissue slices without any cell addition (NT), with UTD T cells or FOLR1 CAR-T cells addition. The areas shown in Figs 4 and 5 are highlighted by the black line. Scale bar: 1 mm. (TIF) [file pone.0327322.s004.tif]
